# Diagnostic value of lymphocyte-to-monocyte ratio in Crohn’s disease: a cross-sectional study

**DOI:** 10.1097/MS9.0000000000003017

**Published:** 2025-02-07

**Authors:** Karwan Anwar Hassan ALJAF, Salih Ahmed Hama, Mohammed Omer Mohammed, Hawbash M. Rahim

**Affiliations:** a Biology Department, College of Science, University of Sulaimani, Sulaimaniyah, Iraq; b Department of Biology, College of Education, Garmian University, Kalar District, Sulaimaniyah, Iraq; c Medical Laboratory Science Department, College of Health Sciences, University of Human Development, Sulaymaniyah, Iraq; d Department of Medicine, College of Medicine, University of Sulaimani, Sulaimani, Iraq

**Keywords:** biomarker, Cohn’s disease, disease activity, lymphocyte-to-monocyte ratio, neutrophil-lymphocyte ratio

## Abstract

**Background:**

Investigating non-invasive laboratory biomarkers for detecting and classifying Crohn’s disease (CD) activity remains challenging. Here, we aimed to evaluate the diagnostic efficacy of immunological biomarkers in identifying disease activity in newly diagnosed CD patients.

**Methods:**

This cross-sectional study was performed from October 2022 to July 2023 and included 20 healthy participants and 46 confirmed newly diagnosed CD patients in a Teaching Hospital for Gastroenterology and Hepatology, Sulaimani, Iraq. The patients were categorized according to the disease activity assessed according to the Harvey-Bradshaw Index and divided into remission and active CD.

**Results:**

A statistically higher serum C-reactive protein (CRP) and erythrocyte sedimentation rate (ESR) levels were found among the remission CD group (*P* = 0.005 and *P* = 0.0002, respectively) and active CD group (*P* < 0.0001 and *P* < 0.0001, respectively) compared to the healthy controls. Moreover, the mean CRP and ESR levels among active CD were also considerably higher than those of remission CD (*P* = 0.018 and *P* = 0.005, respectively). The lymphocyte-to-monocyte ratio (LMR) was significantly lower in patients with active disease (3.01 ± 1.36) than in remission patients (6.77 ± 2.17) (cutoff < 4.42, area under receiver-operating characteristic curve (ROC) = 0.807, 95% CI, 77.35–98.73%, 93% sensitivity, and 78% specificity). Although the neutrophil-to-lymphocyte ratio (NLR) was significantly elevated in active patients (3.64 ± 2.004) compared to healthy controls (1.91 ± 0.48; *P = 0.0001*), it is not usable for disease activity or severity as the area under the ROC curve was 0.68 (95% CI, 52.22–85.08%, sensitivity = 79%, specificity = 62%).

**Conclusion:**

The LMR can be an affordable, easily accessible test that shows promise for determining disease activity in newly diagnosed CD patients.

**HIGHLIGHTS**

- The lymphocyte-to-monocyte ratio was significantly lower in Crohn’s disease patients with active disease than those in remission.
- Neutrophil to lymphocyte ratio cannot serve for disease activity or severity in Crohn disease.
- A positive significant correlation was seen between neutrophil-to-lymphocyte ratio with C-reactive protein and erythrocyte sedimentation rate.
- A negative significant correlation was observed between lymphocyte-to-monocyte ratio with C-reactive protein and erythrocyte sedimentation rate.

## Introduction

Crohn’s disease (CD), a persistent inflammatory ailment, is classified as one of the inflammatory bowel diseases (IBD)^[1,2]^. It is distinguished by inflammation of the gastrointestinal system, although it can potentially impact any region spanning from the oral cavity to the rectum^[3]^. The condition is found worldwide. However, its occurrence differs in different geographical areas. It is predominantly diagnosed in developed nations and has had a growing occurrence in recent years. The annual incidence rate of CD in the Arab world has been estimated to be 1.46/100 000 persons^[4]^. However, there is a lack of data regarding any IBDs in Iraq. Although it can impact people of all age groups, the start of this condition is commonly noted around late adolescence and early adulthood^[5]^.

Diagnosing CD is complicated as it involves a combination of endoscopic imaging, histological examination, and laboratory tests. Endoscopic and biopsy are invasive and limited in use as they are not cost-effective and require sample collection; however, they are still the standard for diagnosing, monitoring, and estimating disease activity^[6]^. Immunological and laboratory tests for identifying CD as non-invasive examinations are still challenging. Routinely, there are laboratory biomarkers for examining inflammation in IBD, including C-reactive protein (CRP) and erythrocyte sedimentation rate (ESR). They are not specific for CD but can monitor the health care for the severity of the disease^[6,7]^.

Differentiation of CD activity using cost-effective biomarkers, easy access, and non-invasive clinical examination has recently been an area of study for many researchers. Recently, researchers have examined numerous blood markers, such as leukocyte differentials, lymphocyte-to-monocyte ratio (LMR), platelet-to-lymphocyte ratio (PLR), and neutrophil-to-lymphocyte ratio (NLR), to determine their usefulness in predicting and assessing the severity of conditions like rheumatoid arthritis, pancreatitis, and some types of cancer^[8-10]^. So far, only a few studies have investigated the usefulness of these ratios in the context of CD, with further studies needed to establish any diagnostic value^[11,12]^. A somewhat recent meta-analysis stated that NLR in peripheral blood may be a useful indicator for assessing disease severity in patients with IBD^[13]^. In ulcerative colitis (UC), Cherfane *et al* and Okba *et al* showed that LMR can be an effective biomarker of disease activity^[14,15]^. An investigation by Nassri *et al* showed that NLR and LMR are ineffective biomarkers in assessing CD disease activity^[11]^. However, Feng *et al* found statistically lower LMR and higher NLR in CD patients than in healthy controls^[12]^.

This study aimed to clarify the diagnostic value of leukocyte ratios in newly diagnosed CD patients compared to healthy controls.

## Materials and methods

### Study design and setting

This was a cross-sectional study that was carried out between October 2022 and July 2023. The study included 20 healthy control volunteers and 46 confirmed newly diagnosed CD patients diagnosed by a professional gastroenterologist at a Teaching Hospital for Gastroenterology and Hepatology, Sulaimani, Iraq. The confirmed diagnosis of CD was based on standard clinical, histological, radiological, and endoscopic evaluations^[16,17]^. Consent was obtained from all participants after providing them with relevant information, and ethical approval was obtained before the initiation of the study. The study has been prepared and reported according to the STROCSS criteria^[18]^.

### Inclusion and exclusion criteria

In the present study, only newly diagnosed CD patients who had not previously received any medications were included because previously diagnosed cases might have received some form of treatment that might influence the biomarkers. Moreover, patients with any other acute or chronic infections or even histories of intestinal surgeries that may possibly influence the leukocytic ratios were excluded.

### Clinical and diagnostic measures

Blood samples were collected from all the included individuals right after confirmed diagnosis by a professional gastroenterologist, which was used for laboratory examinations, such as CRP, ESR, absolute count of neutrophils, lymphocytes, and monocytes using fully automatic five-part hematology analyzer—Medonic M51 (Medonic M51, S. No; TF11041844023, Sweden). The NLR and LMR were also calculated.

According to the Harvey-Harvey-Bradshaw Index (HBI), patients with CD are classified into remission, moderate, and severe based on abdominal pain, overall well-being, fever, diarrhoea, blood per rectum, weight loss, vomiting, and weakness^[19]^. In the current study, besides the control group, newly diagnosed CD patients were subdivided into two groups: active CD group (moderate and severe cases with HBI scores of more than 4) and remission CD group (those with HBI scores of less than or equal to 4).

### Outcomes

The primary outcomes were the absolute counts of neutrophils, lymphocytes, and monocytes, along with the LMR and NLR, as potential biomarkers of disease activity. These parameters were compared across active CD patients, remission CD patients, and healthy controls. The secondary outcomes included the comparison of inflammatory markers such as CRP and ESR between the groups.

### Data collection

Data were collected from each participant, including gender, body mass index (BMI), age at diagnosis, clinical features, and laboratory parameters, including ESR, CRP, neutrophil, lymphocyte, and monocyte counts. The obtained data were entered into Microsoft Excel software.

### Statistical analysis

The statistical analysis was done using GraphPad prism (9.5.1 (528)). The values among CD groups were compared to those of healthy controls. Qualitative variables were analyzed using chi-square (*X*^2^) test. A quantitative analysis was done through one-way ANOVA to compare the three groups. Pearson’s correlation coefficient (Pearson r) was used to assess the correlation between CD disease activity and NLR and LMR. The accuracy of each parameter was assessed by the area under the receiver-operating characteristic (ROC) curve (AUC), and the optimal cutoff point of LMR and NLR was identified with specificity and sensitivity for discrimination of CD’s activation from healthy controls. Statistically significant results were considered when the *P*-value was < 0.05.

## Results

### Characteristics of the participants

In total, the study included 20 healthy controls and 46 confirmed newly diagnosed CD patients (18 with remission CD vs. 28 with active CD). Among the three groups, age and gender were comparable, and no significant difference could be seen (*P* > 0.05). Further characteristics of the enrolled participants and the disease location and phenotype are shown in Tables 1 and 2.

**Table 1:** Characteristics of the study participants.

Characteristics	Healthy control (*n* = 20)	CD-remission (*n* = 18)	CD-active (*n* = 28)	Post hoc		
				*P*_1_	*P*_2_	*P*_3_
Age, mean ± SD	32.15 ± 11.13	31.44 ± 15.81	31.75 ± 13.51	0.87	0.91	0.94
Gender, *n* (%)				0.285		
Male	10 (50%)	12 (66.66%)	12 (42.85%)			
Female	10 (50%)	6 (33.3%)	16 (57.14%)			
BMI (kg/m^2^)	26.9 ± 2.280	23.2 ± 4.41	20.7 ± 4.93	0.0038	<0.0001	0.08
CRP (mg/L)	1.61 ± 0.99	14.82 ± 19.75	31.26 ± 25.7	0.005	<0.0001	0.018
ESR (mm/hour)	8.4 ± 2.87	31.11 ± 20.65	51.39 ± 25.42	0.0002	<0.0001	0.005
Neutrophil (10^3^/μL)	4.508 ± 0.97	4.93 ± 2.17	4.87 ± 1.88	0.45	0.38	0.92
Lymphocyte (10^3^/μL)	2.417 ± 0.5	2.24 ± 1.25	1.59 ± 0.68	0.59	<0.0001	0.053
Monocytes (10^3^/μL)	0.38 ± 0.11	0.39 ± 0.17	0.59 ± 0.26	0.76	0.0004	0.003
NLR	1.91 ± 0.48	2.85 ± 2.37	3.64 ± 2.004	0.11	0.0001	0.25
LMR	6.77 ± 2.17	6.54 ± 4.17	3.01 ± 1.36	0.83	<0.0001	0.0025

P1, remission-CD vs. healthy control; P2, active-CD vs. healthy control; P3, remission-CD vs. active-CD; BMI, body mass index; CRP, C-reactive protein; ESR, erythrocytes sedimentation rate; NLR, neutrophil-to-lymphocytes ratio; LMR, lymphocytes-to-monocytes ratio.

**Table 2:** Location and phenotype of CD among the enrolled participants.

Disease characteristics	Overall (*n* = 46)	CD-remission (*n* = 18)	CD-active (*n* = 28)	*P*-value
Location, *n* (%)				0.696
Ileum (L1)	17 (36.95%)	8 (44.5%)	9 (32.1%)	
Colonic (L2)	12 (26.1%)	4 (22.2%)	8 (28.6%)	
Ileocolonic (L3)	17 (36.95%)	6 (33.3%)	11 (39.3%)	
Phenotype, *n* (%)				0.978
Non-penetrating, non-stricturing (B1)	13 (28.3%)	5 (27.75%)	8 (28.6%)	
Stricturing (B2)	12 (26.1%)	5 (27.75%)	7 (25%)	
Penetrating (B3)	21 (45.6%)	8 (44.5%)	13 (46.4%)	

### Laboratory parameters of the study groups

The laboratory biomarkers for newly diagnosed CD patients were evaluated and compared to healthy controls, as shown in Table 1. The results showed statistically higher serum CRP and ESR among the remission CD group (*P* = 0.005 and *P* = 0.0002, respectively) and active CD group (*P* < 0.0001 and *P* < 0.0001, respectively) compared to the healthy controls. Moreover, the mean CRP and ESR levels among active CD were also considerably higher than those of remission CD (*P* = 0.018 and *P* = 0.005, respectively).

There were no significant differences in absolute neutrophil counts between the groups (*P* > 0.05). Similarly, no considerable differences were also observed in lymphocyte and monocyte counts between controls and remissions (*P* > 0.05). However, considerably lower lymphocyte and higher monocyte counts were seen among the active CD group in comparison to the controls (*P* < 0.0001 and *P* = 0.0004, respectively) and the remission CD group (*P* = 0.053 and *P* = 0.003, respectively).

Active newly diagnosed CD patients had a higher NLR than healthy controls (*P* = 0.0001). However, no significant differences were seen between remission patients and controls or between remission and active CD patients (*P* > 0.05). Although LMR was not significantly different between remission and controls, it was significantly lower among the active CD group in comparison to the controls (*P* < 0.0001) or to the remission CD group (*P* = 0.0025).

### Correlations between leukocyte ratios (LMR and NLR) and disease activity

**Figure 1.**
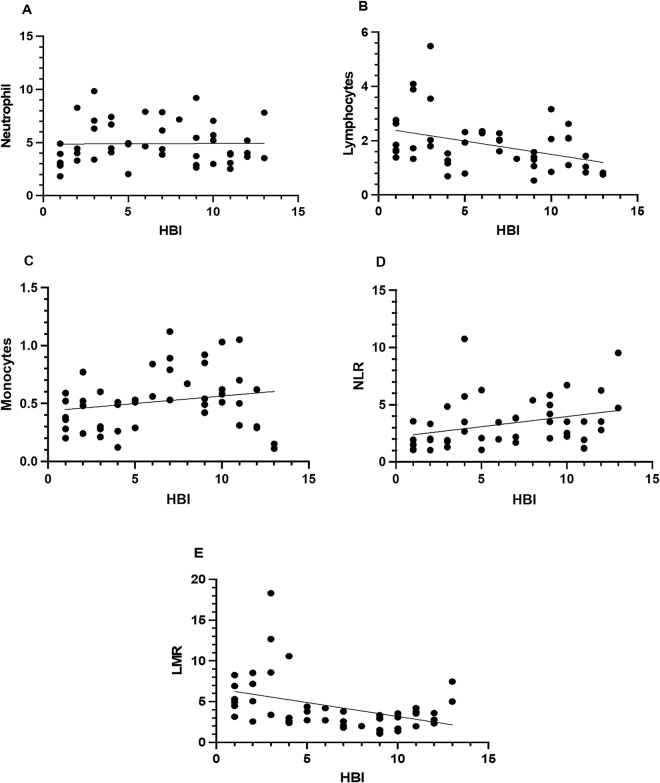
The correlations between the Harvey-Bradshaw Index (HBI) of CD with laboratory blood cells and blood cell ratios.

In the present study, the correlation coefficient between blood biomarkers of leukocyte cells as well as NLR and LMR and the CD disease according to HBI parameters were estimated as shown in Figure 1, and a positive correlation of the neutrophils, monocytes, and NLR was shown (neutrophils, *r* = 0.011, *P* = 0.94; monocytes, *r* = 0.2002, *P* = 0.182; NLR, *r* = 0.318, *P* = 0.0309, respectively). The results showed that the correlation of the lymphocytes and LMR was inverse with HBI (lymphocytes, *r* = − 0.387, *P* = 0.007; LMR, *r* = − 0.406, *P* = 0.005), respectively.

### Correlation of NLR and LMR with other inflammatory markers

**Figure 2.**
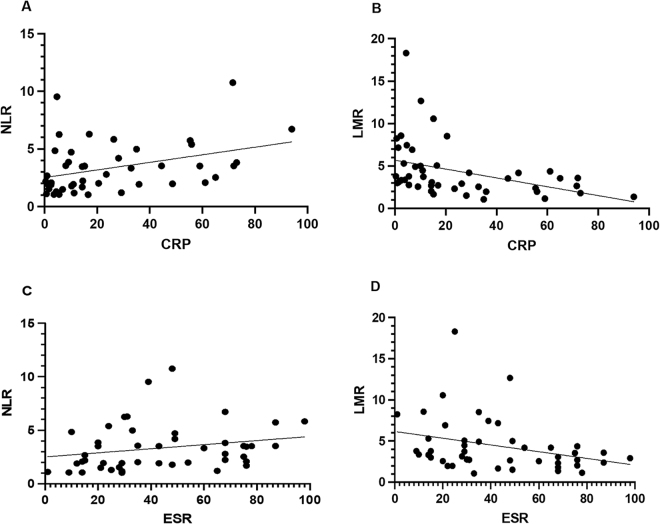
The correlations between the NLR and LMR in CD with laboratory inflammatory biomarkers (CRP and ESR).

The correlation between NLR and LMR with the CRP and ESR was also estimated (Fig. 2), and the results indicated a positive correlation between NLR and CRP (*r* = 0.375, *P* = 0.0102). At the same time, the LMR had a negative correlation (*r* = − 0.391, *P* = 0.007). According to the correlation between NLR and LMR with the ESR, the results indicated the NLR correlation was positive (*r* = 0.22, *P* = 0.13), while LMR had a negative correlation (*r* = − 0.317, *P* = 0.031).

### Biomarker performance for CD activity via ROC analysis

For the identification and differentiation of active CD from remission CD, a ROC analysis was done for the laboratory leukocytes and ratio parameters (Table 3). Surprisingly, the ROC analysis of the LMR gives the best result for differentiating the active from the remission CD (AUC = 0.807, 95% CI, 77.35-98.73%). The cutoff value of LMR was 4.42, with a sensitivity of 93% and a specificity of 78%. NLR gave poor results in disease activity differentiation with an AUC of 0.68 (95% CI, 52.22–85.08%) with a sensitivity of 79% and a specificity of 62%. The ROC analysis of monocytes again gave satisfactory results (AUC = 0.74, 95% CI, 77.35–98.73%). The ROC analysis of CRP and ESR also gave good results (AUC = 0.73, 0.77; 95% CI; 64.41–92.12%, 68.51–94.3%, respectively). In this study, the cutoff values for CRP and ESR were set at 8.5 and 20.5, respectively, achieving a sensitivity of 82% for CRP and 85% for ESR, and a specificity of 56% for CRP and 61% for ESR.

**Table 3:** Evaluation of the diagnostic accuracy of the examined markers in identifying active CD.

Laboratory parameters	Cutoff point	AUC	Sensitivity %	Specificity %
CRP (mg/L)	>8.5	0.73	82	56
ESR (mm/hour)	>20.5	0.77	85	61
Lymphocytes (10^3^/μL)	<2.6	0.63	96	67
Neutrophils (10^3^/μL)	>3.47	0.50	79	68
Monocytes (10^3^/μL)	>0.28	0.74	93	61
NLR	>2.04	0.68	79	62
LMR	<4.42	0.807	93	78

AUC, area under the curve; CRP, C-reactive protein; ESR, erythrocytes sedimentation rate; NLR, neutrophil-to-lymphocytes ratio; LMR, lymphocytes-to-monocytes ratio.

### Performance of LMR combined with inflammatory biomarkers in assessing disease activity

For identification of the efficacy of LMR in combination with inflammatory biomarkers (CRP and ESR), to assess the disease severity, the binary logistic regression was used, in which the disease severity was performed as the independent variable, and the dependent variable was LMR with CRP separately and in combination with ESR. The probability of predicted combinations was then used for the ROC curve scheme. The results indicated that the LMR incorporation with inflammatory biomarkers is good for differentiating the disease severity status, as the ROC analysis of combined LMR with ESR gave the best result (AUC = 0.845, cutoff value = 0.68, sensitivity = 85%, and specificity = 83%, *P*<0.0001), subsequently the incorporation of LMR with CRP and ESR (AUC of 0.833, cutoff value = 0.68, sensitivity = 79%, and specificity = 87%, *P* < 0.0001), and LMR with CRP (AUC = 0.813, cutoff value = 0.56, sensitivity = 93%, and specificity = 69%, *P* = 0.0003) (Table 4).

**Table 4:** Evaluation of the diagnostic accuracy of LMR combined with inflammatory biomarkers (CRP and ESR) in identifying active CD.

Laboratory parameters	Cutoff point	AUC	Sensitivity %	Specificity %
LMR + CRP	0.56	0.813	93	69
LMR + ESR	0.68	0.845	85	83
LMR + CRP + ESR	0.68	0.833	79	87

AUC, area under the curve; CRP, C-reactive protein; ESR, erythrocytes sedimentation rate; LMR, lymphocytes-to-monocytes ratio.

## Discussion

In the current study, we evaluated the CRP, ESR, peripheral blood neutrophils, lymphocytes, and monocytes, as well as the NLR and LMR, to see if they are viable and reliable biomarkers of CD activity. Active CD patients showed significantly higher CRP and ESR levels, elevated monocyte counts, and a higher NLR compared to both controls and remission patients, with LMR being significantly lower in active CD. Correlation analysis found NLR positively associated with disease severity, while LMR was negatively correlated. The ROC analysis highlighted LMR as the best marker for distinguishing active from remission CD.

As the management of IBD advances, biomarkers are used not just for diagnosing and monitoring the condition but additionally for tailoring personalized treatments. The characteristics of an ideal biomarker include being non-invasive, offering high specificity and sensitivity, easy to perform, and cost-effective. Thus far, no biomarkers that fulfill these criteria have been discovered as substitutes for endoscopy^[20]^. Anti-*Saccharomyces cerevisiae* antibodies have been proposed as reliable indicators for managing CD. However, it is worth noting that these antibodies are also commonly observed in individuals with coeliac disease^[21]^. With the exception of CRP and fecal calprotectin, the majority of biomarkers require validation in extensive populations. Still, fecal calprotectin has some limitations, as its utilization in clinical practice is restricted due to its high cost and inconvenient procedures for collecting and processing samples^[22,23]^. CRP and ESR are often employed as inflammatory markers to assess disease activity in individuals with IBD, as also seen in our study. Nevertheless, earlier research yielded unsatisfactory findings due to the limited sensitivity and specificity of CRP and ESR in indicating intestinal inflammation^[24]^. The utilization of CRP or ESR as biomarkers for CD necessitates careful examination, as elevated levels of these markers have also been observed in patients with inflammatory diseases outside of the colon^[25]^.

When comparing our results with those of previous studies, both similarities and differences are observed. A study by Nassri *et al*^[11]^ reported that NLR was ineffective in predicting CD activity. Another study by Xu *et al* revealed that NLR was somewhat related to UC activity but an undesirable marker for CD^[6]^. Cherfane *et al*^[14]^ observed that NLR is effective in distinguishing active UC patients from patients without IBD. A meta-analysis by Fu *et al*^[13]^ found that IBD patients of both CD and UC have significantly elevated NLR than healthy controls. Although they observed that NLR values were higher in active UC compared to remissive UC, the same was not true in the case of CD. Okba *et al*^[15]^ reported NLR as a marker of disease activity. Feng *et al*^[12]^ also identified a statistically significant increase in NLR among CD patients compared to healthy controls, thus highlighting its diagnostic potential. While our study found NLR to be elevated in CD patients, AUC analysis indicated that NLR is not a reliable marker for differentiating between active and remission states of CD. The elevation of NLR in CD may reflect systemic inflammation characterized by neutrophilia and lymphopenia. Neutrophilia is driven by cytokines like IL-6 and TNF-α, while lymphopenia results from chronic immune activation and apoptosis. These processes increase NLR but make it less specific for distinguishing disease activity states.

In regards to LMR, a study by Nassri *et al*^[11]^ reported that LMR was not effective in predicting CD activity, which diverges from our findings where LMR demonstrated significant diagnostic value. Another study by Xu *et al* reveals that LMR is more likely related to UC activity than NLR; however, it was not a desirable marker for CD^[6]^. Although Feng *et al*^[12]^ identified a statistically significant reduction in LMR among CD patients compared to healthy controls, in their study, LMR had no diagnostic utility for disease activity. Further studies focused on only UC rather than CD by Cherfane *et al*^[14]^ and Okba *et al*^[15]^ found that LMR is an effective biomarker for disease activity in UC. This might reinforce the idea that LMR is a broadly applicable marker across different types of IBDs. In the current study, LMR was significantly lower in active CD patients compared to emission CD. The reduction in LMR may be due to monocytosis and lymphopenia in active CD. Monocytosis reflects increased monocyte recruitment to inflamed tissues, while lymphopenia results from immune dysregulation. These changes make LMR a more specific indicator of disease activity than NLR.

This discrepancy in the potential of NLR and LMR might be due to differences in study design and patient population. For instance, our study focused on newly diagnosed and untreated CD patients compared to studies that included patients at various stages of treatment. Moreover, variations in the population’s genetic background, environmental factors, and the various cutoff values used to define active versus remission states might also contribute to these differing results.

In terms of the relationship between NLR and LMR and other inflammatory biomarkers in CD patients, the current study indicated that NLR was positively correlated to CRP and ESR. On the other hand, LMR was negatively correlated to CRP and ESR. These findings are in line with what has been previously reported by Feng *et al*^[12]^ in CD patients and Okba *et al*^[15]^ in UC patients.

Neutrophils are crucial cells that enter the intestinal mucosa during inflammation. Excessive infiltration occurs due to the stimulated recruitment of neutrophils and a malfunction in the process of apoptosis in CD. Therefore, it plays a crucial role in the development of mucosal inflammation observed in IBD. The infiltration of neutrophils into the mucosa aids in the elimination of cellular waste and the eradication of microorganisms that could potentially infect intestinal injuries^[26]^. Neutrophils can also contribute to the process of intestinal healing by stimulating the production of proteins and lipid mediators^[15]^. However, no significant difference in neutrophil count was observed between the groups of this study.

Monocytes and other subtypes of white blood cells have been examined in different inflammatory conditions, such as rheumatoid arthritis and atherosclerosis. In the inflammatory tissues, monocytes develop into dendritic cells and macrophages. They have been identified as biomarkers that can predict the response to treatment or the severity of the disease^[27,28]^. We have demonstrated that an increased number of monocytes at a specific moment is linked to disease activity, consistent with previous research. Nevertheless, more research is needed to examine the significance of peripheral blood monocytes as a long-term prognostic biomarker in individuals with CD^[27,29]^.

## Limitation and prospective

This study has several limitations that should be considered in future research. First, it was conducted in a single center, which may limit the generalizability of the findings. Multi-center studies involving diverse populations would provide more representative results. Second, the small sample size, though reflective of the rarity of the disease, resulted in wide confidence intervals, potentially affecting the precision of the results. Larger cohorts could address this issue in future studies. Third, confounding factors such as diet, smoking, and physical activity, which are known to influence inflammatory biomarkers, were not examined. Future studies should incorporate these variables to provide a clearer understanding of their effects. Additionally, the study focused on systemic markers without exploring localized immune responses, such as those in the gastrointestinal tract. Integrating tissue-based analyses or microbiota profiling could deepen insights into disease mechanisms.

## Conclusion

This study revealed that monocytes and LMR can be indicators of CD activity, with higher monocyte counts in active CD and lower LMR values. An LMR cutoff value below 4.42 effectively distinguishes active CD, demonstrating 93% sensitivity and 78% specificity. The findings suggest LMR could serve as a non-invasive marker for differentiating between active CD and remission in the absence of colonoscopy. However, further research is needed for more accurate results and to solve the disparities among the studies.

## Data Availability

All the data of this study may be provided by the corresponding author upon request.

## Research registration unique identifying number (UIN)

Researchregistry10217.
